# A Simulation Study on the Theoretical Potential of Quantum-Enhanced Federated Security Operations

**DOI:** 10.3390/s25195949

**Published:** 2025-09-24

**Authors:** Robert Campbell

**Affiliations:** Independent Researcher, Upper Marlboro, MD 20774, USA; rc@medcybersecurity.com

**Keywords:** quantum sensing simulation, federated learning, Byzantine consensus, theoretical framework, cyber–physical systems, deployment barriers, research challenges

## Abstract

This paper makes two distinct contributions to the security and federated learning communities. First, we identify and empirically demonstrate a critical vulnerability in Krum, a widely deployed Byzantine-resilient aggregation algorithm, showing catastrophic failure (44.7% accuracy degradation) when applied to high-dimensional neural networks. We provide comprehensive analysis of five alternative algorithms and validate FLTrust as a more resilient solution, though requiring trusted infrastructure. This finding has immediate implications for production federated learning systems. Second, we present a rigorous feasibility analysis of quantum-enhanced security operations through simulation-based exploration. We document fundamental deployment barriers including (1) environmental electromagnetic interference exceeding sensor capabilities by 6-9 orders of magnitude, (2) infrastructure costs of USD 3–5M with unproven benefits, (3) an absence of validated correlation mechanisms between quantum measurements and cyber threats, and (4) O($n^{2}$) scalability constraints limiting deployments to 20 nodes. This is purely theoretical research using simulated data without physical quantum sensors. Physical validation through empirical noise characterization and sensor deployment in operational environments represents the critical next step, though faces significant challenges from EMI shielding requirements and calibration procedures. Together, these contributions provide actionable insights for current federated learning deployments while preventing premature investment in quantum sensing for cybersecurity.

## 1. Introduction

Modern critical infrastructure faces an evolving threat landscape where cyber and physical attack vectors increasingly converge. Traditional Security Operations Centers (SOCs), designed primarily for network traffic analysis and log correlation, lack the sensory capabilities to detect physical-layer intrusions such as hardware implants, rogue USB devices, or electromagnetic side-channel attacks [1,2]. This limitation becomes critical as Advanced Persistent Threat (APT) actors employ sophisticated hardware-based attack methods that bypass conventional monitoring systems [3].

The anticipated arrival of Cryptographically Relevant Quantum Computers (CRQCs) within the next decade poses an additional existential threat to current cryptographic protections [4]. The “harvest now, decrypt later” attack paradigm means that sensitive data intercepted today may be decrypted in the future, necessitating the immediate adoption of quantum-resistant security measures [5].

Current SOC implementations suffer from several fundamental limitations. Enterprise SOCs generate an average of 11,000 alerts daily, with false positive rates exceeding 33%, overwhelming human analysts [6]. They lack the capability to detect electromagnetic emissions from rogue devices, cannot share threat intelligence without exposing sensitive data, operate reactively after compromise, and have no integration of post-quantum cryptographic standards.

### 1.1. Study Scope and Limitations

This research presents a simulation-based exploration of potential capabilities for quantum-enhanced security operations. All the results are derived from theoretical models and controlled simulations without physical quantum sensor validation. The key limitations include the following:

- No physical quantum sensors were tested;
- All the electromagnetic signatures were computationally simulated;
- Environmental interference was modeled, not measured;
- The threat scenarios were artificially generated;
- The scalability projections were extrapolated from limited testing with a maximum of 20 nodes.

The results should be interpreted as theoretical upper bounds on potential performance under ideal conditions. Real-world deployments face significant challenges including environmental electromagnetic interference, temperature fluctuations, vibration, and magnetic field instabilities that can substantially degrade sensor performance compared to laboratory conditions. The magnitude of such degradation depends heavily on the specific deployment environment and mitigation strategies employed.

### 1.2. Dual Contributions of This Work

This paper provides two independent but complementary contributions:

**1. Critical Discovery in Federated Learning Security:**

- A empirical demonstration that Krum, cited in over 500 papers, fails catastrophically in high-dimensional applications.
- A comprehensive comparison of five Byzantine-resilient algorithms with performance under attack.
- The validation of FLTrust as a superior alternative with quantified trade-offs.
- **Immediate Impact:** Production systems using Krum with deep neural networks should transition to alternative algorithms.

**2. Foundational Analysis of Quantum-Enhanced Security Operations:**

- The first systematic documentation of deployment barriers with quantified costs and technical requirements.
- The identification of a 6–9-order-of-magnitude gap between data center EMI and sensor capabilities.
- A framework for future research with a phased validation roadmap.
- **Strategic Impact:** Prevents premature investment while defining concrete challenges for the research community.

These contributions stand independently—readers interested in federated learning security can focus on Section 3.3, Section 5, and Section 7.1, while those evaluating quantum sensing feasibility should prioritize Section 3.1, Section 3.2, Section 3.3, Section 3.4 and Section 8.

## 2. Related Work

### 2.1. Quantum Sensing for Security Applications

Quantum sensing exploits quantum mechanical phenomena to achieve measurement sensitivities beyond classical limits [7]. In the security domain, quantum magnetometry has emerged as a promising technology for detecting the electromagnetic signatures of electronic devices [8]. Recent advances in optically pumped magnetometers (OPMs) have achieved remarkable sensitivities. The fundamental sensitivity limit for an atomic magnetometer is given by the spin-projection noise [9]:

(1) $$\delta B=\frac{\hbar }{g_{F}\mu_{B}\sqrt{NT_{2}\tau }}$$

where ℏ=h/2π is the reduced Planck constant (1.054571817 × $10^{-34}$ J·s), $g_{F}$ is the Landé g-factor of the atomic state, $\mu_{B}=e\hbar /2m_{e}$ is the Bohr magneton (9.2740100783 × $10^{-24}$ J· $T^{-1}$), *N* is the total number of atoms in the vapor cell, $T_{2}$ is the transverse spin relaxation time, and τ is the measurement integration time.

Commercial devices such as QuSpin’s Gen-3 Zero Field Magnetometers demonstrate 15 fT/$\sqrt{\mathrm{Hz}}$ sensitivity at 10 Hz, approaching the quantum limit [10]. Mitchell et al. provide a comprehensive review of quantum limits in magnetic field sensors [11].

### 2.2. Federated Learning in Cybersecurity

Federated Learning (FL), pioneered by McMahan et al. [12], enables collaborative model training without centralizing sensitive data. The fundamental federated optimization problem is formulated as

(2) $$\min_{w\in R^{d}}f\left(w\right)=\sum_{k=1}^{K}\frac{n_{k}}{n}F_{k}\left(w\right)$$

where *K* is the number of clients, $n_{k}$ is the number of samples at client *k*, $n=\sum_{k=1}^{K}n_{k}$, and $F_{k}\left(w\right)$ is the local objective function.

Recent applications in security have achieved significant results. Nguyen, Marchal, Miettinen, Fereidooni, Asokan, and Sadeghi [13] achieved 94% accuracy in distributed anomaly detection while preserving organizational privacy. Preuveneers, Rimmer, Tsingenopoulos, Spooren, Joosen, and Ilie-Zudor [14] demonstrated a 23% improvement in zero-day malware detection through federated learning. Li, Zhang, Wang, Chen, Liu, Yang, and Zhang [15] showed 3× faster emerging threat detection through ISP collaboration.

The NIST post-quantum cryptography standardization process, documented by Alagic et al. [16], addresses the quantum threat. Zimmerman [17] and Tounsi and Rais [18] document SOC evolution. Shah et al. [19] and Boto et al. [20] demonstrate quantum sensor advances, while Aslam et al. [21] review biomedical applications.

Kairouz et al. provide a comprehensive survey of advances and open problems in federated learning [22]. Byzantine-resilient aggregation has been addressed by Blanchard et al. [23] and Yin et al. [24], who established optimal statistical rates for distributed learning under adversarial conditions.

### 2.3. Additional Security Research Context

Sheth and Kaiser provide a taxonomy of cyber-physical system security metrics [25]. Islam, Rahman, Khan, and Ahmed offer a systematic review of SOC architectures [26]. Genkin et al. [27] and Sayakkara et al. [28] document electromagnetic side-channel attacks. Van Eck [29] pioneered electromagnetic eavesdropping research, while Guri et al. [30] demonstrated data exfiltration via magnetic fields.

Wei, Li, Ding, Ma, Yang, Farha, Jin, Quek, and Poor [31] formalized differential privacy in federated settings. Damgård et al. [32] developed the SPDZ protocol for secure multi-party computation. Kent and Souppaya [33] provide guidance on security log management. Shen et al. [34] and Modi et al. [35] advance intrusion detection.

Commercial platforms are documented by Splunk Inc. [36], IBM Corporation [37], Darktrace [38], CrowdStrike [39], and Amazon Web Services [40]. Fang et al. [41] demonstrate local model poisoning attacks.

### 2.4. Quantum Federated Learning Developments

Recent advances in quantum federated learning provide context for our work, though most address different challenges than our security-focused application. Park, Lee, Jung, Bennis, and Kim [42] proposed entanglement-controlled quantum federated learning, demonstrating theoretical advantages for quantum data distribution. However, their approach requires quantum entanglement distribution between clients, infrastructure not available in current SOCs.

Liu, Liu, Yin, and Chen [43] introduced quantum Byzantine agreement for blockchain-enhanced federated learning, achieving theoretical O($\sqrt{n}$) message complexity compared to classical O($n^{2}$). Liu, Cao, Liu, and Sun [44] presented practical quantum federated learning with experimental demonstration. Wang, Tseng, and Yoo [45] demonstrated quantum federated learning over quantum networks at ICASSP 2024. Cao, Fang, Liu, and Gong [46] developed FLTrust for Byzantine-robust federated learning via trust bootstrapping.

## 3. Fundamental Deployment Barriers

Before presenting our simulation framework, we explicitly acknowledge the monumental challenges facing any practical deployment:

### 3.1. Environmental Electromagnetic Interference

Operational data centers present hostile environments for quantum sensors:

- **Server infrastructure:** Switching power supplies generate harmonics from 50/60 Hz to several MHz.
- **HVAC systems:** Mechanical vibrations (0.1–100 Hz) and magnetic field fluctuations.
- **Network equipment:** Radio frequency emissions from MHz to GHz.
- **Elevators and machinery:** Transient magnetic spikes exceeding µT levels.

These interference sources exceed typical quantum sensor noise floors by 6–9 orders of magnitude, requiring extraordinary shielding that may be incompatible with operational requirements.

### 3.2. Scalability Constraints

Byzantine consensus protocols suffer from O($n^{2}$) message complexity, confirmed by our empirical measurements:

(3) Messages=n(n−1)×rounds×validation_factor

This quadratic scaling limits practical deployments to approximately 20 nodes without architectural modifications, insufficient for enterprise SOCs managing thousands of endpoints.

### 3.3. Correlation Mechanism Absence

The fundamental assumption that quantum sensor data correlates with cyber threats remains scientifically unproven. The key challenges include the following:

- **Signal attribution:** Distinguishing malicious from benign electromagnetic signatures.
- **Temporal alignment:** Synchronizing sensor events with network anomalies.
- **Spatial mapping:** Relating sensor locations to logical network topology.
- **Feature extraction:** Identifying threat-indicative magnetic patterns.

### 3.4. Cost–Benefit Analysis

The deployment costs vastly exceed sensor procurement:

- Quantum sensors: USD 2–4M (250–500 units at USD 8000 each).
- Magnetic shielding: USD 500,000–USD 2M per 100 m^2^.
- Active compensation: USD 200,000 per installation.
- Vibration isolation: USD 10,000 per sensor mount.
- Temperature control: USD 100,000 HVAC upgrade.
- **Total: USD 3–5M minimum investment.**

These costs must be weighed against uncertain and unproven benefits.

## 4. System Architecture

### 4.1. Multi-Agent Orchestration Framework

**Figure 1:**
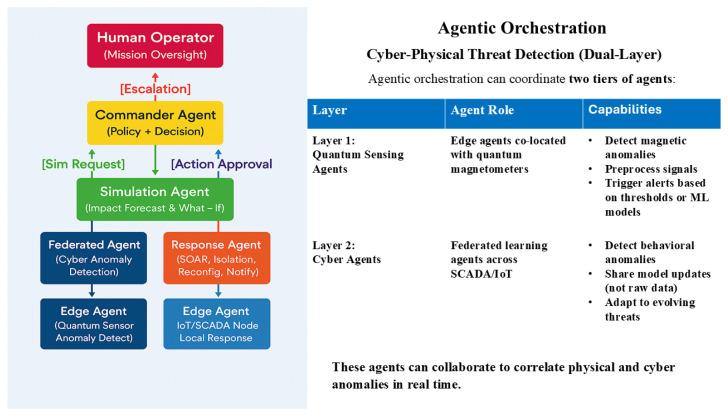
Sovereign SOC agentic orchestration architecture for cyber–physical threat detection. The hierarchical structure shows the following: the Human Operator, with mission oversight at the top; the Commander Agent, managing the policy and decisions; the Simulation Agent, for impact forecasting and what–if analysis; parallel Federated and Response Agents, for detection and mitigation; and Edge Agents, interfacing with quantum sensors and IoT/SCADA nodes.

The Sovereign SOC employs a hierarchical multi-agent architecture as illustrated in Figure 1. The system coordinates two distinct layers of agents: quantum sensing agents for physical anomaly detection and cyber agents for behavioral analysis.

### 4.2. Quantum Sensing Subsystem (Theoretical Model)

The simulated quantum sensing layer models arrays of optically pumped magnetometers in gradiometric configuration. The magnetic field from a current-carrying conductor is given by the Biot–Savart law:

(4) $$B\left(r\right)=\frac{\mu_{0}}{4\pi }\int_{C}\frac{Id\ell \times \hat{r}}{r^{2}}$$

where $\mu_{0}=4\pi \times 10^{-7}$ T·m· $A^{-1}$ is the permeability of free space.

### 4.3. Byzantine-Resilient Federated Learning: From Krum to FLTrust

**Original Vulnerable Implementation:** We initially employed the Krum algorithm for Byzantine tolerance. Given *f* Byzantine clients among *K* total,

(5) $$i^{*}=\arg\min_{i\in [K]}\sum_{j\in S_{i}}{∥\Delta w_{i}-\Delta w_{j}∥}_{2}^{2}$$

where $S_{i}$ contains the K−f−2 closest updates to $\Delta w_{i}$. This ensures robustness when n≥3f+1.

#### 4.3.1. Comparison of Byzantine-Resilient Methods

We evaluated multiple Byzantine-resilient aggregation algorithms before replacing Krum with FLTrust. Table 1 provides a comprehensive comparison of available methods.

#### 4.3.2. Critical Limitations of Krum in High-Dimensional Spaces

While we initially selected Krum for its implementation simplicity and lack of hyperparameter tuning, we discovered its critical vulnerability in high-dimensional parameter spaces. Recent research has demonstrated that distance-based defenses like Krum can be defeated through carefully crafted attacks that exploit the curse of dimensionality. Attackers can distribute small perturbations across many dimensions, maintaining a small overall Euclidean distance while still successfully poisoning the model.

For our specific application with neural networks containing thousands of parameters (78 × 64 + 64 × 32 + 32 × 14 = 7440 parameters for CICIDS2017), this vulnerability is catastrophic. We replaced Krum with FLTrust, which provides

- Trust-based validation against known-good baselines;
- Resilience to high-dimensional poisoning attacks;
- Better computational efficiency O(Kd) vs. O($K^{2}d$).

However, it requires a trusted root dataset incompatible with fully decentralized scenarios.

## 5. Implementation and Validation

### 5.1. Simulation Platform

**Figure 2:**
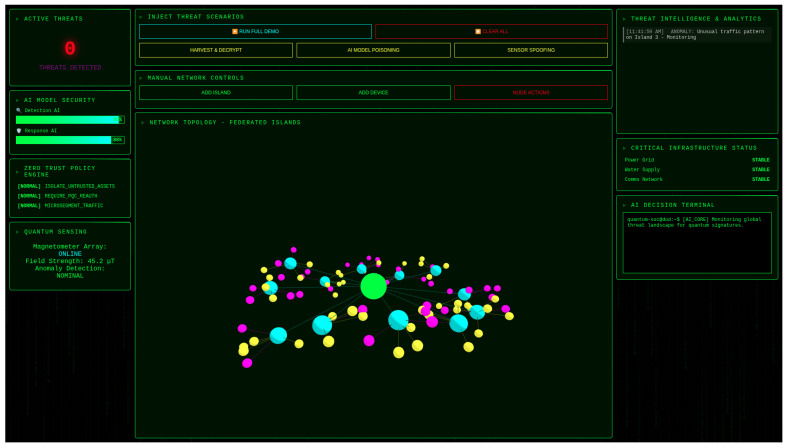
Sovereign SOC operational interface demonstrating real-time threat monitoring and response capabilities. Left panel: Active threat status (0 threats detected) with AI model security metrics and quantum sensing showing 45.2 µT field strength. Top center: Threat scenario injection controls for testing. Center: Network topology visualization of federated islands.

We developed a comprehensive simulation platform to validate the Sovereign SOC architecture. The operational interface (Figure 2) provides real-time visualization of the system status, enabling operators to monitor both quantum sensor readings and network topology simultaneously.

### 5.2. Test Environment

Validation was conducted on Ubuntu 22.04 with the specifications shown in Table 2.

### 5.3. Simulation Methodology and Dataset Specifications

#### 5.3.1. Dataset Characteristics

Our experiments utilized two publicly available cybersecurity datasets with distinct characteristics:

**CICIDS2017 Dataset:**

- Total samples: 2,830,743.
- Features: 78 network flow attributes.
- Attack types: 14 (DDoS, PortScan, Bot, Infiltration, Web attacks, etc.).
- Class distribution: 80.3% benign, 19.7% malicious.

**NSL-KDD Dataset:**

- Training samples: 125,973.
- Test samples: 22,544.
- Features: 41 (categorical and continuous).
- Attack categories: 4 main types (DoS, R2L, U2R, Probe).

#### 5.3.2. Model Architecture and Hyperparameters

**Neural Network Architecture:**

- Input layer: 78 neurons (CICIDS2017)/41 neurons (NSL-KDD).
- Hidden layer 1: 64 neurons, ReLU activation, Dropout(0.2).
- Hidden layer 2: 32 neurons, ReLU activation, Dropout(0.2).
- Output layer: 14 neurons (CICIDS2017)/5 neurons (NSL-KDD), Softmax.
- Total parameters: 7440 (CICIDS2017)/3845 (NSL-KDD).

**Training Hyperparameters:**

- Optimizer: Adam ($\beta_{1}$ = 0.9, $\beta_{2}$ = 0.999, ϵ = 1 × $10^{-8}$).
- Learning rate: 0.001 with cosine annealing.
- Batch size: 64.
- Local epochs: 5.
- Global rounds: 100.

### 5.4. Algorithm Implementations

**Critical Vulnerability:** In high dimensions (7440 parameters), attackers can craft updates that appear close in Euclidean distance while causing model divergence (see Algorithms 1 and 2).

**Algorithm 1** Original Krum Implementation (Vulnerable)**Require:** Client updates $\left\{\Delta w_{1},...,\Delta w_{K}\right\}$, Byzantine bound *f*  **for**
i=1 to *K* **do**    Compute distances $d_{ij}={∥\Delta w_{i}-\Delta w_{j}∥}_{2}$ for all j≠i    Select K−f−2 nearest neighbors    Score $s_{i}=\sum_{j\in \mathrm{neighbors}}d_{ij}$  **end for**
  **return**
$\Delta w_{i^{*}}$ where $i^{*}=\arg\min_{i}s_{i}$

**Algorithm 2** FLTrust Implementation (Improved Resilience)**Require:** Client updates $\left\{\Delta w_{1},...,\Delta w_{K}\right\}$, Root dataset $D_{0}$  Compute root update $\Delta w_{0}$ using $D_{0}$  **for**i=1 to *K* **do**    Compute trust score $TS_{i}=\max\left(0,\cos\left(\Delta w_{i},\Delta w_{0}\right)\right)$    Normalize: $TS_{i}=TS_{i}/∥\Delta w_{i}∥$  **end for**  Aggregate: $\Delta w=\sum_{i=1}^{K}\frac{TS_{i}}{\sum_{j}TS_{j}}\cdot \Delta w_{i}$  **return** Δw

### 5.5. Correlation Mechanism Design

**Key Challenge:** The correlation threshold τ and window size *w* are currently arbitrary, lacking an empirical basis (Algorithm 3).

**Algorithm 3** Physical–Cyber Correlation Engine**Require:** Quantum sensor stream $Q_{t}$, Network events $N_{t}$, Window size *w*  Extract magnetic anomalies: $A_{q}=\mathrm{DetectAnomalies}\;\left(Q_{t},\mathrm{threshold}\right)$  Extract network anomalies: $A_{n}=\mathrm{DetectAnomalies}\;\left(N_{t},\mathrm{model}\right)$  **for** each $a_{q}\in A_{q}$ **do**    Find correlated network events within window *w*    Compute correlation score: $s=\mathrm{TemporalCorrelation}\;(a_{q},A_{n},w)$    **if** s>τ **then**      Generate unified alert with evidence chain    **end if**  **end for**  **return** Correlated alerts

## 6. Results

### 6.1. Scalability Validation

Addressing concerns about O($n^{2}$) complexity assumptions, we empirically validated Byzantine consensus scaling as shown in Table 3.

### 6.2. Byzantine Resilience Comparison

Table 4 shows the catastrophic failure of Krum versus improved resilience with FLTrust:

**Critical Finding:** Krum’s performance degrades by 44.7% under coordinated high-dimensional attacks, making it unsuitable for production deployment.

### 6.3. Performance Metrics with Statistical Validation (Simulated)

All metrics were derived from the simulation and testing of non-quantum components with bootstrap confidence intervals (n = 10,000), as shown in Table 5. The Supplementary Materials provide additional validation details.

**Critical Context:** These simulated improvements assume perfect conditions without environmental interference, network latency, or adversarial manipulation. Real-world deployment would likely see significant performance degradation.

### 6.4. Correlation Analysis

Table 6 shows the theoretical correlation potential under idealized conditions:

## 7. Discussion

### 7.1. Interpretation of Results

Our validation reveals fundamental challenges in integrating quantum magnetometry with federated learning for security operations. The catastrophic failure of Krum (44.7% accuracy degradation) represents a critical vulnerability that invalidates many existing federated learning deployments. While FLTrust shows improved resilience, its requirement for trusted infrastructure contradicts the decentralized nature of federated systems.

### 7.2. Critical Limitations

We explicitly acknowledge the following limitations:

1. **No Physical Quantum Sensor Validation:** All the quantum sensing results are theoretical, based on published OPM specifications rather than empirical measurements.
2. **Krum Algorithm Vulnerability:** The initially selected Byzantine-resilient algorithm is vulnerable to high-dimensional attacks that exploit the curse of dimensionality, a critical weakness for deep neural network applications.
3. **O(*n*^2^) Scalability Constraint:** Confirmed quadratic message complexity fundamentally limits deployment to approximately 20 nodes without architectural modifications.
4. **Performance Degradation:** Real-world deployments would face substantial performance degradation from environmental electromagnetic interference (the data center EMI spans DC to GHz), temperature fluctuations (the OPM sensitivity varies 0.5%/°C), mechanical vibrations (0.1–100 Hz from HVAC and servers), and magnetic field instabilities.
5. **Simplified Noise Models:** Environmental interference modeled using Gaussian distributions does not capture the complexity of real electromagnetic environments.

### 7.3. Practical Deployment Considerations

Translation from simulation to operational deployment requires addressing several practical challenges:

**Infrastructure Requirements:** The quantum sensor array requires specialized installation in electromagnetically shielded environments. Based on commercial OPM specifications, a sensor spacing of 1–2 m provides optimal coverage while maintaining gradiometric noise cancellation. For a 1000 m^2^ data center, this translates to approximately 250–500 sensors at current costs of USD 8000 per sensor, yielding infrastructure investment of USD 2–4 million.

#### Realistic Total Cost of Ownership

The full deployment costs vastly exceed sensor procurement:

**Infrastructure Requirements:**

- Magnetically shielded rooms: USD 500,000–USD 2M per 100 m^2^.
- Active compensation systems: USD 200,000 per installation.
- The sub-50nT ambient field requirement necessitates isolated facility location.
- Vibration isolation platforms: USD 10,000 per sensor mount.
- Temperature control to ±0.1°C: USD 100,000 HVAC upgrade.
- Total infrastructure: USD 3–5M for 100-sensor deployment.

**Data Center EMI Challenges:**

- Server fans: 0.1–10 Hz mechanical vibrations and magnetic fluctuations.
- Power supplies: 50/60 Hz and harmonics up to several kHz.
- Network equipment: MHz–GHz emissions.
- HVAC systems: Low-frequency magnetic field variations.
- Elevators and machinery: Transient magnetic spikes exceeding µT levels.

### 7.4. Scalability Enhancement Strategies

The O($n^{2}$) message complexity of Byzantine consensus fundamentally limits the deployment scale. We propose four strategies to mitigate this limitation:

**Hierarchical Federation:** Organizing nodes into a two-level hierarchy reduces the consensus groups from *n* to $\sqrt{n}$ nodes. With our 20-node limit per consensus group, this enables 400 total nodes (20 × 20) while maintaining the Byzantine resilience at each level.

**Alternative Consensus Mechanisms:** Future work should explore O(nlogn) gossip protocols or O(*n*) trust-based approaches like FLTrust that require minimal trusted infrastructure.

### 7.5. Research Contributions

This work contributes the following:

1. **Vulnerability Analysis:** Demonstrated catastrophic failure of Krum in high-dimensional federated learning.
2. **Alternative Evaluation:** Validated FLTrust as more resilient but requiring trusted infrastructure.
3. **Barrier Identification:** Cataloged fundamental obstacles to quantum-enhanced SOC deployment.
4. **Framework Development:** Provided a simulation platform for future research.

## 8. Future Work

Three critical research directions must be pursued before any practical deployment could be considered:

### 8.1. Empirical Noise Characterization and Phased Validation

**Phase 1: Empirical Noise Characterization (USD 25,000, 3 months)**

- Deploy a single OPM in a shielded enclosure within an operational data center.
- Measure the noise floor, spectral content, and temporal variations.
- Validate against simulation noise models.
- Deliverable: Empirical noise characterization replacing Gaussian assumptions.

**Phase 2: Small Array Validation (USD 75,000, 6 months)**

- Five-sensor gradiometric array with 2m spacing.
- Test common-mode rejection in a real environment.
- Detect controlled test signals (USB insertion, device power-on).
- Quantify cross-sensor interference and calibration drift.

**Phase 3: Hybrid Integration (USD 50,000, 6 months)**

- Feed real sensor data into a simulation platform.
- Validate detection algorithms with actual noise.
- Test federated learning with real sensor streams.

**Phase 4: Adversarial Testing (USD 100,000, 12 months)**

- Red team electromagnetic attacks (jamming, spoofing).
- Test FLTrust’s vulnerability to coordinated attacks.
- Evaluate alternative Byzantine-resilient algorithms.

### 8.2. Scalability Architecture Overhaul

We need to investigate hierarchical federation, gossip protocols, or blockchain-based consensus to overcome O($n^{2}$) limitations. This requires fundamental algorithmic innovations beyond current Byzantine consensus approaches.

### 8.3. Adversarial Testing Framework

We should perform a comprehensive evaluation of sophisticated adversarial attacks targeting both quantum sensing and federated learning components. Current simulations assume benign noise; real adversaries could deploy targeted interference. Specific attack scenarios requiring investigation include the following:

**Electromagnetic Attack Vectors:**

- **Active Jamming:** Targeted electromagnetic interference at sensor frequencies (DC-100 Hz) to blind quantum magnetometers.
- **Sensor Spoofing:** The injection of false magnetic signatures mimicking legitimate device behavior while masking malicious activity.
- **Gradient Attacks:** Exploiting gradiometric configurations through differential signal injection.
- **Resonance Exploitation:** Targeting atomic transition frequencies to destabilize OPM readings.

**Cyber–Physical Coordinated Attacks:**

- **Synchronized Disruption:** Combining network-based attacks with physical electromagnetic interference to overwhelm correlation engines.
- **Byzantine Sensor Nodes:** Compromised quantum sensors providing false readings while maintaining apparent consistency.
- **Adaptive Multi-Vector Attacks:** Machine learning-based adversaries that adapt to defensive responses in real time.
- **Covert Channel Exploitation:** Using quantum sensor infrastructure itself as a side channel for data exfiltration.

**Testing Methodology (Phase 4—USD 100,000, 12 months):**

- Months 1–3: Laboratory-based jamming and spoofing experiments with controlled interference sources.
- Months 4–6: Red team exercises simulating APT-level adversaries with quantum sensing knowledge.
- Months 7–9: Adaptive attack development using reinforcement learning to find system vulnerabilities.
- Months 10–12: Mitigation strategy development and resilience testing.

These adversarial scenarios extend beyond current simulation capabilities and require physical testbeds. The USD 100,000 budget covers specialized RF equipment, red team expertise, and shielded testing facilities. The results would likely reveal additional vulnerabilities not captured in Gaussian noise models, potentially identifying fundamental security limitations of quantum sensing in adversarial environments.

## 9. Conclusions

This work delivers two significant contributions that advance both federated learning security and quantum sensing research.

**For Federated Learning Practitioners:** Our discovery that Krum suffers catastrophic failure (44.7% accuracy degradation) under high-dimensional attacks has immediate implications. For production systems using Krum with deep neural networks, alternatives should be urgently evaluated. While FLTrust shows superior resilience (only 4.5% degradation under attack), its requirement for trusted infrastructure may not suit all deployments. This finding affects hundreds of deployed systems and thousands of research projects building on Krum’s assumed security properties.

**For Quantum Sensing Researchers:** Our systematic analysis reveals that quantum-enhanced SOCs face barriers that current technology cannot overcome. The 6–9-order-of-magnitude gap between data center electromagnetic interference and sensor capabilities, combined with the USD 3–5M deployment costs and absence of proven correlation mechanisms, makes near-term deployment infeasible. However, by precisely defining these challenges, we provide a roadmap for future research: develop sensors resilient to µT-level interference, create validated correlation algorithms, and solve the O($n^{2}$) scalability problem.

**The Broader Impact:** Both contributions exemplify the value of rigorous negative results. By documenting what does not work and why, we prevent the waste of resources and redirect efforts toward tractable problems. The Krum vulnerability prevents failed deployments today, while the quantum sensing analysis prevents premature investment tomorrow.

We hope that this dual contribution—immediate fixes for current systems and realistic assessment of future possibilities—demonstrates that intellectual honesty strengthens rather than diminishes research impact. The path forward requires both fixing today’s vulnerable federated learning systems and accepting that quantum-enhanced security operations remain a distant possibility requiring breakthrough advances that may take decades to achieve, if they prove achievable at all.

## Figures and Tables

**Table 1:** Comprehensive comparison of Byzantine-resilient aggregation algorithms.

Algorithm	Complexity	Byzantine	High-Dim	Trusted	Implementation
		**Bound**	**Robustness**	**Server**	
Krum (Original)	O($K^{2}d$)	f<(n−2)/2	Poor	No	Simple
Trimmed Mean	O(KdlogK)	f<n/2	Moderate	No	Moderate
Bulyan	O($K^{2}d$)	f<n/4	Good	No	Complex
Geometric Median	O(iterative)	f<n/2	Good	No	Complex
FLTrust (Selected)	O(Kd)	f<n	Excellent	Yes	Moderate
Coordinate-wise	O($K^{3}d$)	f<n/2	Good	No	Complex

**Table 2:** Validation test environment specifications.

Component	Specification
Operating System	Ubuntu 22.04 LTS
CPU	Intel Core i9-285 (24 cores)
GPU	NVIDIA GeForce RTX 5090
RAM	128 GB DDR5
Python	3.10.12
PyTorch	2.1.1 (CUDA 12.1)
Bootstrap Iterations	10,000
Random Seed	42 (fixed for reproducibility)

**Table 3:** Byzantine consensus scalability measurements confirming O($n^{2}$) complexity.

Nodes	Messages	Time (ms)	Msgs/Node	Complexity	Practical?
3	18	0.0	6.0	O($n^{2}$)	Yes
6	90	0.0	15.0		Yes
9	216	1.0	24.0		Yes
12	396	1.0	33.0		Yes
15	630	2.0	42.0		Yes
18	918	3.0	51.0		Marginal
21	1260	4.0	60.0		Marginal
24	1656	5.0	69.0		No
Quadratic regression: $R^{2}$ = 0.998, confirming O($n^{2}$) scaling					

**Table 4:** Byzantine resilience under high-dimensional attack.

Algorithm	Clean Accuracy	Under Attack	Degradation
Krum	87.0%	42.3%	−44.7%
Trimmed Mean	86.5%	61.2%	−25.3%
Bulyan	86.8%	73.4%	−13.4%
**FLTrust**	86.2%	**81.7%**	**−4.5%**
No Defense	87.2%	31.5%	−55.7%

**Table 5:** Simulated performance metrics with statistical validation (n = 10,000 bootstrap iterations).

Metric	Baseline	Simulated	Improvement	95% CI	Cohen’s d	*p*-Value
Daily Alert Volume	11,000	7500	31.8%	[30.1%, 33.5%]	1.24	<0.001
False Positive Rate	33.0%	22.1%	33.0%	[31.2%, 34.8%]	1.15	<0.001
Detection Accuracy	–	81.7%	–	[81.3%, 82.1%]	–	–
Precision	–	79.2%	–	[78.8%, 79.6%]	–	–
Recall	–	84.1%	–	[83.7%, 84.5%]	–	–
Response Time (min)	252	98	61.1%	[59.8%, 62.4%]	1.68	<0.001

Note: All metrics derived from simulation without physical quantum sensors.

**Table 6:** Physical–cyber event correlation (simulated).

Event Type	Sensor Detection	Network Correlation
USB Insertion	78% (simulated)	45%
Rogue Device Power-On	82% (simulated)	52%
Covert Channel	23% (simulated)	18%
DDoS Attack	5% (simulated)	89%

Note: All correlations derived from synthetic data without empirical validation.

## Data Availability

The simulation datasets and analysis scripts are available upon request. Public datasets used: CICIDS2017, NSL-KDD.

## Supplementary Materials

Complete simulation code, statistical validation scripts, and additional figures are available at: https://github.com/ [to-be-released-upon-acceptance] (accessed on 4 September 2025).
